# Landscape predictors of human–leopard conflicts within multi-use areas of the Himalayan region

**DOI:** 10.1038/s41598-020-67980-w

**Published:** 2020-07-07

**Authors:** Dipanjan Naha, Suraj Kumar Dash, Abhisek Chettri, Pooja Chaudhary, Gaurav Sonker, Marco Heurich, Gopal Singh Rawat, Sambandam Sathyakumar

**Affiliations:** 1 Department Endangered Species Management, Wildlife Institute of India, Chandrabani, Dehradun, Uttarakhand India; 2 Large Mammal Ecology Group, University of Freiburg, Freiburg im Breisgau, Germany

**Keywords:** Ecology, Zoology, Ecology

## Abstract

Conflict with humans is a significant source of mortality for large carnivores globally. With rapid loss of forest cover and anthropogenic impacts on their habitats, large carnivores are forced to occupy multi-use landscapes outside protected areas. We investigated 857 attacks on livestock in eastern Himalaya and 375 attacks in western Himalaya by leopards between 2015 and 2018. Multivariate analyses were conducted to identify the landscape features which increased the probability of livestock depredation by leopards. The risk of a leopard killing livestock increased within a heterogeneous landscape matrix comprising of both closed and open habitats (very dense forests, moderate dense forests, open forests, scrubland and non-forests). We used the results to map potential human–leopard conflict hotspots across parts of the Indian Himalayan region. Our spatial risk maps indicate pockets in the eastern, central and western part of eastern Himalaya and the central, northern part of western Himalaya as hotspots of human–leopard conflicts. Most of the attacks occurred when livestock were grazing freely within multi-use areas without supervision of a herder. Our results suggest that awareness about high risk areas, supervised grazing, and removing vegetation cover around human settlements should be initiated to reduce predation by leopards.

## Introduction

Large carnivores are apex predators and their lethal and non-lethal effects have strong implications for ecosystem structure and functioning. They also have prominent cultural reverence in several human societies and act as flagship species for global conservation campaigns^1^. In areas where their populations have suffered local extinction, cascading effects have been observed on ecosystem processes either mediated by abundance or behavior of mesocarnivores and prey^2, 3^. Low population densities, high energetic requirements, social or solitary hunting strategies and wide-ranging behavior means that they often range beyond nature reserves for access to space and resources and seek out easy prey i.e. livestock and occasionally humans within shared landscapes. Species commonly involved in predation on livestock are canids (*Canis* spp.), felids (*Panthera* spp.) and ursids (*Ursus* spp.) across both Northern and Southern Hemispheres^4^. Government agencies, wildlife managers and conservationists spend substantial money to compensate losses to carnivores through a combination of preventative, assertive and reactive measures^5, 6^. In areas where the perceived risk of large carnivores is high, attacks on humans and livestock lead to retaliation by the local communities increasing their extinction risk^3^, and a setback for conservation efforts^7^.

Though protected areas are essential for conserving large carnivores, their size is often not large enough to sustain carnivore populations or even individual home ranges^8^. Therefore, the quality of the larger multi-use landscapes in terms of the availability of suitable habitat, abundance of wild prey, extent of human presence and tolerance of local communities determine future survival of these species. Such shared landscapes represent a major proportion of the geographic distribution of large carnivores globally^9^. Increasing human populations, declines in wild prey and changes in land use patterns have resulted in fragmented, heterogeneous resource limited landscapes for carnivores^10^. Such anthropogenic impacts on ecosystems have forced carnivores to frequent areas near human settlements, kill livestock and consequently local communities have retaliated through poisoning, shooting, snaring and electrocution^11–13^. A substantial proportion of large felid mortality globally (cheetah, eurasian lynx, tiger, jaguar and snow leopard) has been an outcome of human–carnivore conflicts^10^. Subsequently, livestock depredation has been identified as the stimuli for human–carnivore conflicts^10^. Retaliation also affects the overall functioning of ecosystem by removal of non-target species (avian predators, scavengers and mesocarnivores through poisoning of carcasses) and larger predators within the shared landscapes^14^. Conservation within shared landscapes is thus challenging especially considering the socio-cultural, political problems where it is crucial to ensure safety of human lives, property, livelihoods and well-being.

One practical approach used to mitigate human–carnivore conflicts has been to identify the spatial extent of risk zones^15^ and use it in developing conservation policies and planning interventions. In areas where large carnivores co-occur with humans, landscape features have been used as a proxy to determine their presence and model risk of predation on livestock^16–18^. Carnivores have been documented to repeatedly kill prey in areas with easy accessibility and similar landscape features. Such landscape features are a mosaic of several attributes (habitat type, prey density, human activity, availability of water)^16, 19, 20^. Composition and arrangement of these variables in the landscape directly impact hunting success, favoring attributes where prey is easy to detect, catch or encounter thus making certain areas hotspots of conflicts^19, 21, 22^. Wolves killed prey in relatively flat areas with the probability of risk being highest in scrubland, pastures and sites located at increasing distance from dense forests^15, 23^. Hyenas killed prey in areas with dense vegetation cover^17^. Predation by lions were higher within riverine, closed habitats and the vicinity of protected reserves^11, 17, 24, 25^. Puma, jaguar and tiger were documented to kill livestock in areas with dense vegetation cover^26–29^. Altitude also influenced predation risk with pumas and jaguars killing more prey in elevated mountainous regions^26, 27^ whereas lions, leopard and hyenas preferred flat, low lying areas^25^. Human infrastructure such as distribution, length and distance to roads, towns, villages and night light also influenced predation risk by large carnivores^23, 28, 29^. Apart from landscape features, human–carnivore conflicts are often a consequence of both human and carnivore behavior. Poor guarding practices, location of grazing pastures close to protected reserves, and lack of animal shelters also impact the extent of predation on livestock^10^^.^

Leopards are the most resilient of large felids, yet habitat loss and persecution by humans have resulted in significant reductions of their populations with only 17% of the present distribution range protected^30^. India has a sizeable population of leopards and they share space with humans within the majority of the agro-pastoral, forested landscapes^31^. Human–leopard interactions are especially intense within such shared landscapes where the size of protected areas is small, fragmented and comprise only 5% of the geographic extant of the country. Livestock and free ranging domestic dogs comprise the primary prey for leopards^32, 33^ outside of protected areas where crop fields, tea plantations provide cover^34–36^. Predation on livestock has been recognized as a major conservation problem for the species^37–39^ leading to increasing retaliation by the local communities. Domestic dogs and livestock also act as major attractants for leopards near human habitations and attacks on humans often occur as a consequence of leopard presence near settlements^32, 33, 40^. Livestock are a direct representation for the agro-pastoral societies of rural India and loss to large carnivores represents a substantial threat to human welfare and livelihoods. Hence it is crucial to identify areas where leopards are more likely to attack livestock to plan management interventions and implement mitigation measures.

Spatial risk modelling has been widely used to provide insights into habitats where carnivores kill livestock through both qualitative and graphical guides and help in prioritization of management interventions and mitigation measures^15^. This is essentially a quantitative tool which uses location-specific data (kills) to predict the probability of a predator attack by relating landscape attributes at kill sites and comparing them to random sites representing landscape availability. Risk models help in quantifying the realized predation risk (where livestock or wild prey were killed) especially across heterogeneous landscapes^16, 22^. We use a similar multivariate analytical approach to model risk of livestock depredation by leopard within the Indian Himalayan Region (IHR). We investigate patterns of leopard predation on livestock, analyze site specific conditions and map conflict hotspots. Specifically, we examine (1) extent of livestock depredation by leopard in IHR, (2) identify animal husbandry practices which increase vulnerability of livestock to leopard attacks, (3) identify high predation-risk zones based on landscape features within the human-dominated landscapes of IHR, (4) spatially map human–leopard conflict hotspots by modelling probability of livestock predation. Finally, we calculate (5) threshold values for important landscape features and use the results to recommend strategies to reduce human–leopard conflicts within IHR.

## Results

### Extent of livestock predation by leopards

In 2015–2018, there was a total of 857 and 375 leopard livestock predation events recorded in North Bengal and Pauri Garhwal, respectively. 54% of the livestock killed were adult cows, 16% were goats, 14% calves, 6% adult pigs, 5% piglets, 3% ox, 2% goat yearlings in North Bengal (χ^2^_=_ 312.12, df = 7, *p* < 0.05). In Pauri Garhwal, 70% percent of the livestock predated were adult cows followed by 9% adult goats, 8% cattle calves, 7% ox, 1% horse, 2% donkey, 2% sheep and 1% mules (χ^2^_=_ 208.28, df = 8, *p* < 0.05).

### Characteristics of kill sites and animal husbandry practices

In North Bengal livestock killing was recorded along the foothills with an average elevation of 269 m (SE 192) (range 80–2,400) whereas they were recorded higher up at an average altitude of 1,271 m (SE 218) range (540–2,135) in Pauri Garhwal. An average of 2 houses with a range of (0–12) were present within the vicinity (50-m radius) of livestock predation sites in North Bengal. In Pauri Garhwal, an average of 3 houses range (0–30) were present within the vicinity of predation sites. Based on the vegetation types recorded within a diameter of 100 m of the conflict site, the majority of these predation events (37%) were recorded in tea gardens, 17% in scrublands, 13% in sal dominated patches, 13% in horticulture plantations, 5% in teak dominated and 4% within bamboo plantations in North Bengal. In Pauri Garhwal they were recorded in (48%) the vicinity of villages, 41% with moderate vegetation cover, 7% at roadsides and only 3% within livestock sheds. 95% and 41% of the livestock killed in North Bengal and Pauri Garhwal were unsupervised.

### Seasonal and temporal patterns of livestock predation

Livestock predation peaked in the winter (November-February, 42%) and spring (March-June, 37%) seasons, whereas it was lower during monsoons (21%) in North Bengal (χ^2^_=_ 8.676, df = 2, *p* < 0.05). In Pauri Garhwal, the majority occurred in the summer (March-July, 45%) and winter (November–February, 31%) seasons and only 24% during monsoons (March-June) (χ^2^_=_ 6.1987, df = 2, *p* < 0.05). 53% of the predation events were recorded during the day (8AM–4PM), 40% between (12AM–8AM) and the rest 7% between (4PM–12PM) in North Bengal (χ^2^_=_ 33.034, df = 2, *p* < 0.05). 48% of the predation events were recorded during day (4PM–12AM), 46% between (8AM–4PM) and rest 6% between (12AM–8AM) in Pauri Garhwal (χ^2^_=_ 33.68, df = 2, *p* < 0.05).

### Influence of landscape features on leopard attacks on livestock

Moran’s I identified spatial clusters of livestock predation within both North Bengal and Pauri Garhwal. The z value (13.422), Moran’s Index (0.395) and (*p* value < 0.01) indicate that there was a less than 1% likelihood that this pattern was due to random chance in North Bengal. The threshold distance between each neighboring livestock depredation site was estimated to be 5,000.50 m.

Results of the GLM model with binomial structure suggests that landscape features such as area of non-forest (β = 7.81E−08; 95% CI = 3.62E−09–1.4082E−07), scrubland (β = 1.88E−06; 95% CI = 7.7064E−07–2.98936E−06), open forests (β = 2.56E−07; 95% CI = 1.8152E−07–3.30284E−07), very dense forests (β = 1.98E−07; 95% CI = 1.13328E−07–2.82672E−07), area of water (β = − 1.61E−06; 95% CI = 5.0848E−07–2.71E−06), distance to protected areas (β = − 1.70E−04; 95% CI = 0.0001–0.0002), nightlight (β = − 7.45E−02; 95% CI = 0.0137–0.1352) and altitude (β = − 5.26E−03; 95% CI = 0.0033–0.0072) were the best predictors for leopard attacks on livestock in North Bengal (Table 1, Supplementary Table S1). Leopards were more likely to kill livestock within both closed (dense forests) and open habitats (non-forest, open forest and scrubland) in North Bengal. Probability of livestock killing decreased with increasing distance from protected areas. In addition, livestock killing was more likely to happen in flat low lying areas with less human presence and low availability of water. Results of the GLM model with poisson structure are similar and suggests that landscape features such as area of very dense forests (+), open forests (+), scrub (+), area of water (−), distance to protected areas (−) and altitude (−) were best predictors of livestock predation (Supplementary Table S2). Probability of livestock killing were higher within sites with dense to moderate vegetation cover and decreased with human presence, increasing distance form protected areas and water bodies.

**Table 1:** Second-order Akaike Information criterion scores (AIC), (AICc), ΔAICc of generalized linear models with binomial structure predicting livestock depredation by common leopards in North Bengal landscape, using landscape predictors.

Model	AIC	AICc	ΔAIC	BIC
Altitude + nightlight + distance from PAs + area of water bodies + area of non-forest + area of scrub + area of open forest + area of very dense forest	361.01	361.32	0	400.59
Altitude + nightlight + distance from PAs + length of roads + area of water bodies + area of non-forest + area of scrub + area of open forest + area of very dense forest	362.56	362.93	1.61	406.55
Altitude + nightlight + distance from PAs + area of water bodies + area of scrub + area of open forest + area of very dense forest	365.41	365.65	4.33	400.59
Altitude + nightlight + distance from PAs + area of water bodies + area of open forest + area of very dense forest	376.41	376.59	15.27	407.20
Altitude + nightlight + distance from PAs + area of open forest + area of very dense forest	378.37	378.51	17.19	404.76
Altitude + distance from PAs + area of open forest + area of very dense forest	380.68	380.77	19.45	402.67
Distance from PAs + area of open forest + area of very dense forest	412.90	421.96	60.64	439.49
Distance from PAs + area of open forest	427.01	427.05	65.73	440.21
Distance from PAs	467.03	467.05	105.73	475.83

*PAs* protected areas.

The z value (11.329), Moran’s Index (0.499) and (*p* value < 0.01) indicate that there was less than 1% likelihood that this pattern was due to random chance in Pauri Garhwal. The threshold distance between each neighboring livestock depredation site was estimated to be 5,077.024 m.

Results of the GLM model with binomial structure suggests that landscape features such as area of moderate dense forests (β = 2.95E−07; 95% CI = 1.21E−07–4.70E−07), open forests (β = 4.62E−07; 95% CI = 2.27E−07–6.97E−07), scrub (β = 5.58E−07; 95% CI = 8.30E−08–1.03E−06), non-forests (β = 3.38E−07; 95% CI = 1.92E−07–4.85E−07) and the distance to protected areas (β = 3.11E−05; 95% CI = 7.83E−065.44E−05) were the best predictors of livestock predation by leopard in Pauri Garhwal (Table 2, Supplementary Table S3). The probability of livestock killing increased within moderate to open habitats and with distance to protected areas. Results of the GLM model with poisson structure suggests that landscape features such as area of moderate dense forests (+), open forests (+), scrubland (+), non-forest (+), water (−), distance to protected areas (+) and night light (+) were the best predictors for leopard attacks on livestock (Supplementary Table S4). The probability of livestock killing increased within moderate to open habitats. In addition, livestock killing was most likely to occur in areas with less water, more human presence and increasing distance from protected areas.

**Table 2:** Second-order Akaike Information criterion scores (AIC), (AICc), ΔAICc of generalized linear models with binomial structure predicting livestock depredation by common leopards in Pauri Garhwal landscape, using landscape predictors.

Model	AIC	AICc	ΔAIC	BIC
Distance from PAs + area of non-forest + area of scrub + area of open forest + area of moderate dense forest	231.62	231.95	0	254.84
Distance from PAs + area of water bodies + area of non-forest + area of scrub + area of open forest + area of moderate dense forest	232.58	233.03	1.08	257.34
Nightlight + distance from PAs + area of water bodies + area of scrub + area of open forest + area of moderate dense forest	232.80	233.39	1.44	261.10
Nightlight + distance from PAs + length of river + area of water bodies + area of non-forest + area of scrub + area of open forest + area of moderate dense forest + area of very dense forest	232.82	233.72	1.77	268.19
Nightlight + distance from PAs + length of roads + length of rivers + area of water bodies + area of non-forest + area of scrub + area of open forest + area of moderate dense forest + area of very dense forest	234.56	235.65	3.7	273.47
Distance from PAs + area of non-forest + area of open forest + area of moderate dense forest	235.5	235.75	3.8	253.19
Area of non-forest + area of open forest	247.08	247.18	15.23	257.69
Distance from PAs + area of non-forest + area of open forest	247.44	247.60	15.65	261.59
Area of open forest	288.59	288.64	56.69	295.67

*PAs* protected area.

**Figure 1:**
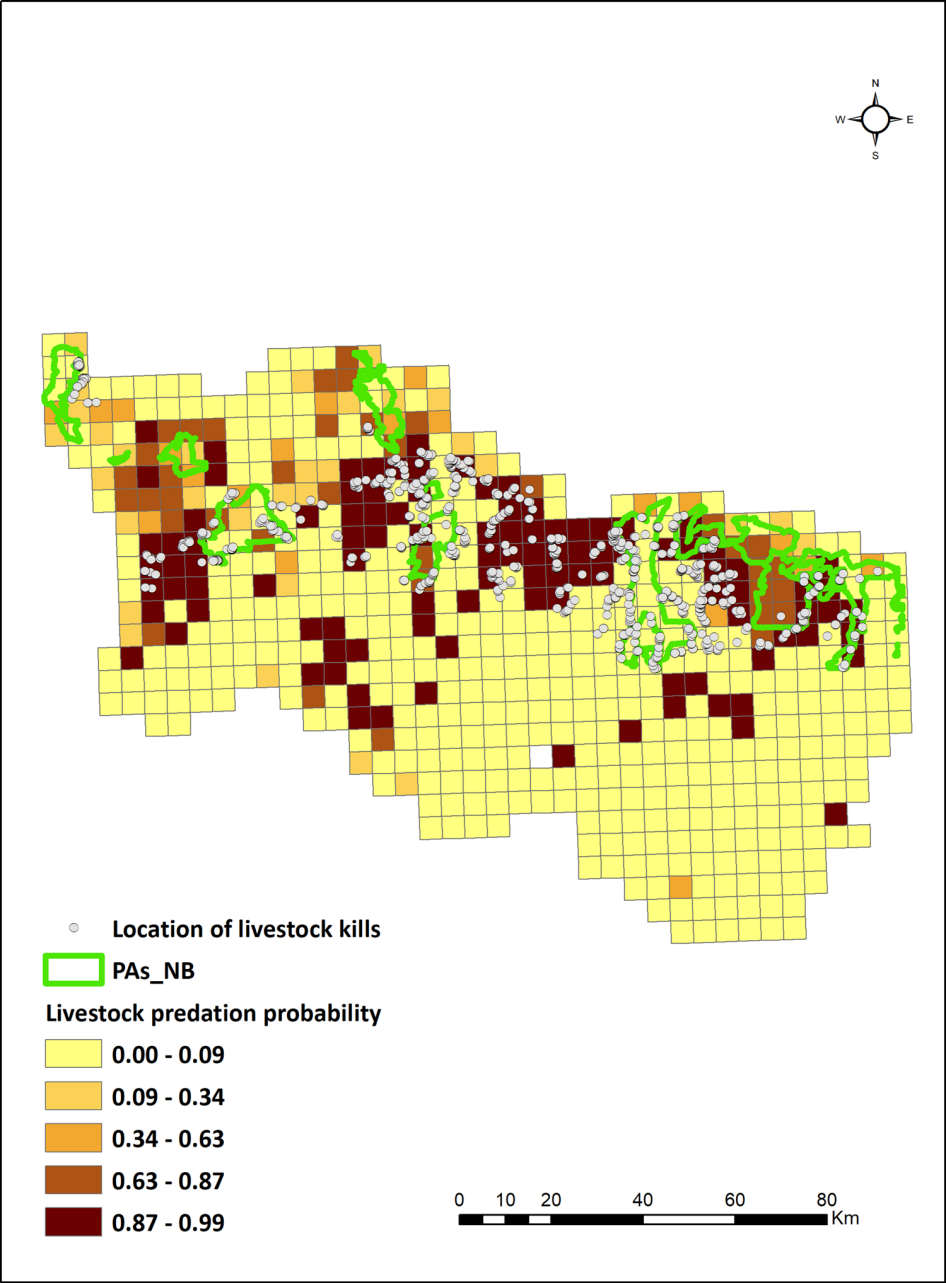
Leopard livestock predation risk map, North Bengal prepared using Arc GIS 10.3 (https://enterprise.arcgis.com/en/portal/10.3/use/link-to-items.htm).

**Figure 2:**
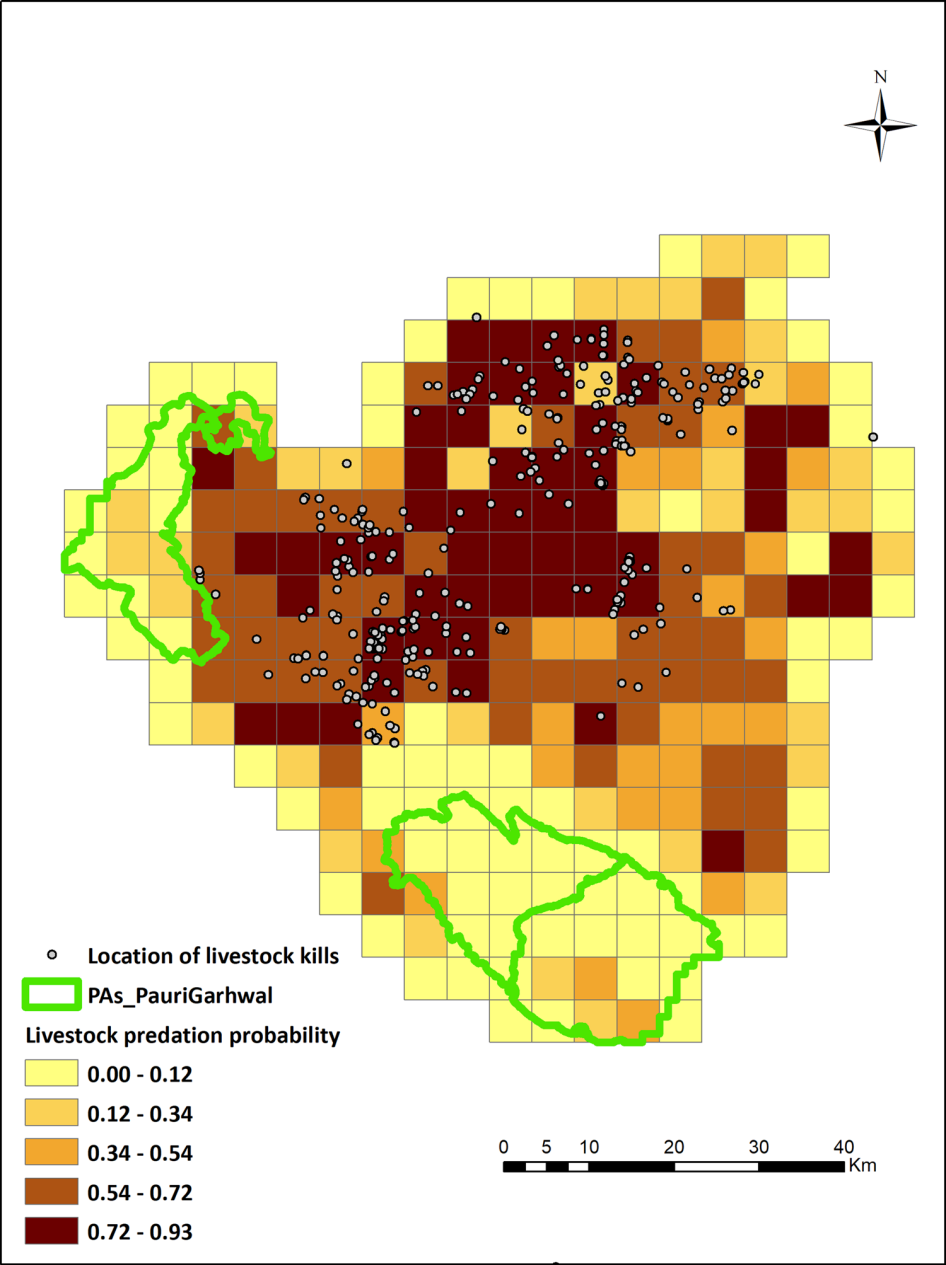
Leopard livestock predation risk map, Pauri Garhwal prepared using Arc GIS 10.3 (https://enterprise.arcgis.com/en/portal/10.3/use/link-to-items.htm).

Receiver operating curves (AUC) values for the dominant model (GLM with binomial structure) were estimated to be 0.86 and 0.83 respectively for North Bengal and Pauri Garhwal. The predictive maps based on the model averaged coefficients indicated that certain pockets within eastern, central, and western parts of North Bengal were hot spots of livestock predation for leopards (Figs. 1, 2). For Pauri Garhwal, predation risk was high in the central and north eastern part of the landscape. Conflict probabilities were higher near protected areas in North Bengal whereas they were lower in Pauri Garhwal.

Wilcoxon rank sum test results indicate that majority of the predictor variables (area of very dense forests, area of moderate dense forests, area of open forests, area of scrub, area of water/riverine patches, area of non-forest, length of roads, length of river, nightlight, *p* < 0.05) differed significantly between the two study sites (Supplementary Table S5). Only distance to protected areas between the two study sites did not exhibit a statistically significant difference (*p* > 0.05).

### Variable thresholds

The results of the conditional inference trees revealed significant thresholds for four variables regarding predation on livestock, i.e. distance from protected areas, altitude, area of open and very dense forests for North Bengal landscape. For Pauri Garhwal landscape, area of non-forested regions (i.e. human settlements) and area of open forests were identified within a landscape scale of 25 km^2^. Multivariate trees were found with a first split indicating highest predation probability (> 0.85) when distance from protected areas (≤ 6,385 m), altitude (≤ 193 m) and intermediate probability (0.80) when area of open forests (i.e. tea plantations) was greater than 7,430,000 sq. meters i.e. 30% for North Bengal. For Pauri Garhwal, predation risk was highest in areas (> 0.86) when area of non-forests was less than 6,820,000 m^2^ i.e. 27% and area of open forests was greater than 3,630,000 i.e. 15%. These results indicate a first priority for foothills, a distance of 6.3 km from protected areas, dense forests (dense vegetation cover) and open forests (tea plantations) for predation risk by leopards in North Bengal. For Pauri Garhwal landscape, risk of predation was highest in areas within a matrix of non-forests (human settlements) and open forests (low vegetation cover).

## Discussion

Our results suggest that landscape features are major predictors of livestock predation by leopards in IHR. Our study also highlights that there is significant spatial clustering of livestock kills by leopards within multi-use landscapes of IHR. At a coarser spatial scale, livestock depredation risk was higher within both closed and open habitats in North Bengal (very dense forests, open forests, non-forests, scrub). In Pauri Garhwal risk was higher within a matrix of moderate and open habitats (moderate dense forests, open forests, scrub and non-forests). Generally, predation risk probabilities by large carnivores are reported to increase with dense vegetation cover^16, 24, 41^ but our results suggest that risk of livestock predation by leopards are higher within a multi-use matrix of open and closed habitats. Our results also indicate that the hunting behavior of leopards and choice of livestock kill sites are different compared to the other large carnivores who rely on dense vegetation cover. Such hunting strategies are probably an artifact of the adaptive nature of leopards, their ability to survive within close proximity of human settlements and consume a wide range of prey^30, 32, 42^. Human–carnivore conflicts have been generally reported along the periphery of protected areas^8, 10^, and probability of livestock killing by leopards increased with proximity to protected areas in North Bengal and decreased with increasing distance from such reserves in Pauri Garhwal. Through this study we also mapped human–leopard conflict hotspots within the IHR which should be prioritized by protected area managers, district administration, local communities and multi-interest groups.

Large carnivore species e.g., hyenas^43^, brown bears^44^, lions^45^ and tigers^29^ use protective vegetation cover to hunt both wild and domestic prey. In contrast, leopards are reported to kill wild prey within moderate vegetation cover such as open mixed woodlands^21^ in Africa whereas in India availability of water was a crucial spatial driver of leopard kills^46^. Our result is similar to a study conducted in the Himalayan region of Bhutan which documented that livestock predation by leopards was higher within a matrix of forest and agriculture^47^. Leopards are stalk and ambush predators frequenting the edge of protected reserves^30, 48^ and heterogeneous landscapes (settlements, grazing lands, interspersed with moderate forests) might offer better prey catchability compared to dense closed habitats. Vegetation cover is an essential requirement for large carnivores and open forests and scrubland interspersed within forested areas in North Bengal provide ideal refuges for leopards. Such vegetation cover is similar to sugarcane, coffee plantations and agricultural fields which have been identified as major drivers of human–leopard conflicts within fragmented habitats of South Asia^35, 36^. As reported by an earlier study, leopard attacks on humans in Pauri Garhwal were negatively associated with the presence of dense forests^36^ but more influenced by a matrix of agriculture lands, shrub cover and medium dense forest patches. Within a fine scale, availability of water has often been identified as a major driver of human–carnivore conflicts in arid ecosystems of Africa^25, 41, 49^ but within productive ecosystems of South Asia availability of water is probably not a limiting factor.

The threshold value of 6.3 km adjoining protected areas in North Bengal is similar to previous studies which documented livestock predation risk to be high within 5–12 km of the park boundary in Nepal and Cameroon^43, 44^. In South Africa risk of a leopard killing livestock was highest within the periphery of protected reserves^50^. Though the probability of livestock killing increased at the edge of protected reserves, we document contrasting results from the two landscapes. Such results could be due to interspecific competition between two large cats^25, 39^ within the same landscape i.e. tiger and leopard. Pauri Garhwal has two protected areas in the foothill region of the district with a sizeable population of Bengal tigers^51^ and due to competitive exclusion leopards probably are more widely distributed in the hills away from the reserve boundaries and hence conflicts with leopards are higher as we move farther away from the reserves. North Bengal region has no other apex predators to compete with leopards (due to recent extinction of tigers) and thus human–leopard conflicts are confined to the edge of protected areas and decrease with increasing distance from such reserves. Thus, patterns of conflicts with the species show a different spatial pattern between the two sites.

Leopards are reported to kill livestock in rugged areas of Bhutan^47^ but our results suggest that livestock kills occurred within flat low lying and mid elevated zones. In North Bengal the average elevation of sites where livestock were killed was low i.e. 270 m probably as a consequence of abundant livestock availability in the foothills compared to the hills. In Pauri Garhwal, the average elevation of livestock kill sites was 1,200 m indicating higher livestock availability in the middle Himalayas compared to the foothills^52^.

There was a strong effect of seasonality on the number of attacks with the majority occurring in the dry season (winter and summer). These results are in accordance with studies conducted in the Terai region of Nepal^53^ where leopard attacks on livestock were also reported to be higher in the dry season (winter and summer months). Our results are contrary to studies conducted in East Africa where predation on livestock by lions, hyenas and leopards were much higher in the wet season compared to the dry seasons^54, 55^. In Africa, wild prey availability is low during the wet season^55^ whereas in South Asia there is probably no significant association of wild prey and seasonality. The seasonal pattern of predation events further coincides with harvesting and planting of major agricultural crops such as maize, wheat and paddy. During these dry months, local community members are often not available to guard livestock. Livestock grazing is higher during winter within agricultural fields, tea plantations and scrubland due to unavailability of forage within the forest interiors.

Livestock kills within both sites were both diurnal and nocturnal which differs with similar studies conducted in the Himalayan region i.e. Pakistan^56^ and Bhutan^57^ where leopard attacks were nocturnal in nature. Though leopards have been reported to be nocturnal in areas with and without humans^48, 58^ our results suggest diurnal activity peaks within human dominated landscapes of the Himalayan region. Similar to leopards, cheetahs also exhibit diurnal activity peaks within human-dominated landscapes of eastern Africa^59^. Big cats have generally been reported to kill livestock at night as with jaguars^60^ in the Pantanal, lions, leopards in Africa^61^ and tigers in India^62^. Leopards probably hunt wild prey at night but livestock killing is more pronounced during the day due to availability and ease of prey catchability.

Leopards in general are reported to kill cattle, goats and sheep when wild prey biomass falls below 540 kg/km^2^^63^. Limited studies on leopard diet within anthropogenic landscapes of India highlight the major contribution of livestock^32, 33^. Prey abundance and availability is a major driver of human–carnivore conflicts^63^ but landscape level data on prey abundance was not available for the study sites to confirm such a relationship. However, our results indicate that wild prey biomass could be low in the Indian Himalayan region leading to increased attacks on livestock. Cattle loss to leopard attacks was higher in Pauri Garhwal whereas predation on goats were higher in North Bengal. Cattle are largely left unsupervised due to the low economic benefits compared to goats in Pauri Garhwal and hence predation on cows were higher. In North Bengal, cattle provide greater economic benefits and hence are generally supervised by an experienced herder. Cattle density (indigenous and cross breed) was also high i.e. 66 animals per km^2^ whereas goat density was 27 animals per km^2^ in Pauri Garhwal (Livestock Census 2012). Cattle density in North Bengal was 92 animals per km^2^ whereas goat density was 80 animals per km^2^ (Livestock Census 2012). Leopards show preference for wild prey species with an estimated prey weight range (10–40) kg^11, 54, 55^, and hence exhibit similar size preference when preying on livestock. Thus, goats were killed in higher abundance in North Bengal compared to Pauri Garhwal.

Previous studies in the mountainous regions of South Asia have documented leopard attacks on livestock to be geographically widespread without much significant association with human presence^57, 64^. In Bhutan livestock predation probability at a fine scale by leopards and tigers were positively associated with the density of human settlements^47^. Our results suggest that at a wider landscape scale, human presence (indicated by night light) is negatively and positively related to probability of conflicts in North Bengal and Pauri Garhwal, respectively. North Bengal is a densely populated region with human densities ranging from 200–700 individuals per km^2^ whereas in Pauri Garhwal, human density is much lower i.e. 110 individuals per km^2^. Leopards probably are adapted to co-occur close to humans but there exists a threshold beyond which there is an underlying avoidance and could be a result of carnivore’s perception of fear (persecution risk) within multi-use landscapes^65^. Our results are similar to space use by lions and cheetahs who avoided humans at a landscape scale due to the fear of persecution^59, 66^.

Output of the risk models is generally used globally to focus interventions and plan implementation of mitigation measures for minimizing damage caused by human–carnivore conflicts^15, 16^. The conflict risk maps generated through this study will be helpful to prioritize mitigation measures and reduce livestock depredation by leopards within IHR. Such measures will help reduce the present extent of retaliation by local communities and ensure survival of leopards outside of protected reserves. Policy makers will be able to better allocate resources to compensate livestock losses, forest and wildlife administration will be able to concentrate mitigation measures within specific sites and local communities will be able to avoid grazing of livestock within high risk zones. Livestock depredation can be reduced by improving existing animal husbandry practices such as using trained livestock guardian dogs and professional herders while grazing. To reduce economic damage to leopard attacks, agro-pastoralist societies of the IHR should be provided with monetary incentives through community based nature tourism initiatives. Awareness programs should be organized to educate livestock owners about biology of leopards, high risk areas and patterns of human–leopard conflicts. Radio-telemetry studies should be initiated to understand fine scale habitat utilization by leopards within anthropogenic landscapes. Finally, collaborations between managers, conservationists, local communities and understanding cultural complexities of human–leopard relations will be crucial to ensure future coexistence within heterogeneous mountainous landscapes of South Asia.

## Methods

### Study area

The study was conducted across two landscapes (North Bengal and Pauri Garhwal) located in eastern and western Himalaya.

**Figure 3:**
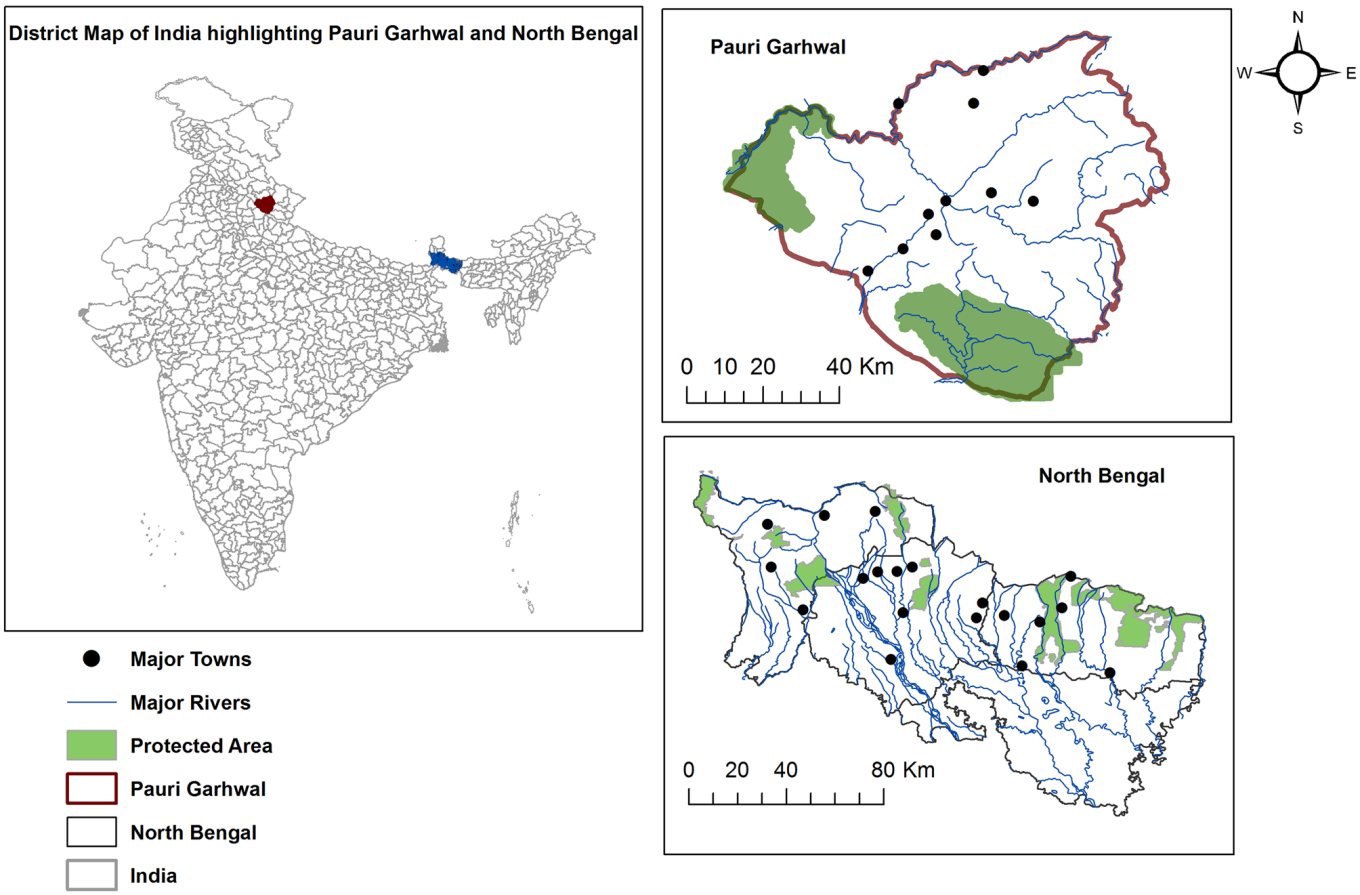
Location of North Bengal and Pauri Garhwal within India along with protected areas and major rivers prepared using Arc GIS 10.3 (https://enterprise.arcgis.com/en/portal/10.3/use/link-to-items.htm).

The North Bengal landscape is located in the north-eastern part of India (Fig. 3) with a forest cover of 46%^67^. Predominant forest types are northern tropical semi-evergreen, moist deciduous forest and the landscape is a matrix of tea gardens, protected areas, agricultural lands and urban settlements^59^. Altitudinal variation is between 50 – 3,500 m with an annual rainfall range of 1,200–3,200 mm. Human density varies between 200 and 700 persons per km^2^ (Census 2011, https://www.census2011.co.in/census/district/1-darjiling.html. Accessed October 2019, https://www.census2011.co.in/census/district/2-jalpaiguri.html. Accessed October 2019, https://www.census2011.co.in/census/district/3-koch-bihar.html. Accessed October 2019) with the primary occupation being livestock farming, agriculture and tea industry^68^. Livestock density is about 340 per km^2^^33^. Major wild prey is sambar (*Rusa unicolor*), chital (*Axis axis*), rhesus macaque (*Macaca mulatta*), and barking deer (*Muntiacus muntjak*). There has been the recent extinction of Bengal tiger (*Panthera tigris tigris*) from this region with leopard (*Panthera pardus fusca*) being the apex predator and only large carnivore present^69^.

Pauri Garhwal district, part of the lesser, middle Himalaya and located in the north-western part of India (Fig. 3) has an altitudinal range of (200–3,200 m) with a forest cover of 64%^67^. Predominant forest types are moderate dense forests, open forest and scrubland^58^. Annual rainfall range is between 1,000 and 2,500 mm. Human density is 110 persons per km^2^ (Census 2011, data accessed on July 2019) and the local inhabitants are mainly agrarian with livestock farming, horticulture and cottage industries as major professions^64^. Livestock density of this region is 58 per km^2^ (Livestock Census, 19th All India Livestock Census Report 2012). This district has two protected areas in the foothill region with a sizeable population of Bengal tiger (*Panther tigris tigris*)^51^. Other than tigers, common leopards are the second most dominant apex predator in this region with presence of other mammals such as barking deer (*Muntiacus muntjak*), goral (*Naemorhedus goral*), sambar (*Rusa unicolor*), wild pig (*Sus scrofa*), rhesus macaques (*Macaca mulatta*) and common langur (*Semnopithecus entellus*).

### Data collection

We used a two-step technique to collect data on leopard predation on livestock. First, we searched newspapers and West Bengal, Uttarakhand state forest department compensation registers for reports of incidents of leopard attacks on livestock in North Bengal and Pauri Garhwal between 2015 and 2018. Our primary aim was to avoid strong spatial bias in data and hence we checked for incidents which were spatially spread out and not confined to specific localities or regions. The compensation registers contained information such as date of incident, carnivore species involved, number and type of livestock killed and name of village/locality. Second, we conducted structured interviews across the regions and asked the owners and community members about age and species of livestock killed by leopards (survey protocol, see Supplementary Information Appendix 1). The livestock owner and forestry officials who were present during the attack or had verified the authenticity of the incident escorted the research team during such field visits. If there was ambiguity in response for a particular incident, we refereed only to the compensation register detail. We also crosschecked every detail of the incident with the report submitted by forestry officials after the initial investigation. The forestry officials were responsible for evaluation of wildlife attacks which is the basis for payment of compensation payments. To avoid exaggeration of livestock losses, we informed the community members that there will be no incentive or compensation provided as a part of the survey and the results will be solely used to help demarcate high and low conflict risk zones. We also recorded season and a time to livestock killed by leopards and asked owners about how often shepherds or community members used to accompany the animals during grazing (Supplementary Information Appendix 1). Based on the information gathered, we visited 857 sites in eastern Himalaya (North Bengal) and 375 sites in western Himalaya (Pauri Garhwal) where leopards had killed livestock. All field visits were made by the research team between November 2017 and November 2019. The forestry department officials accompanied the research team on all field visits and interviews. Ethical approval was obtained from the Wildlife Institute of India and office of the Chief Wildlife Wardens of Uttarakhand and West Bengal for conducting interviews. All methods and experiments (non-medical) were carried out in accordance with the relevant guidelines and regulations of the Declaration of Helsinki. Informed consent was obtained from all subjects or their parents, legal guardians if they were below the age of 18 years. The locations visited were later mapped with a spatial accuracy of 20–50 m. To better understand the circumstances of attacks, we recorded all major habitat types and number of houses present within a radius of 50 m of each site.

### Data analysis

Since precise timing for livestock predation was not known we used coarser 8 h intervals to assign correct time of the depredation events. Each 24-h period was subdivided into 8-h intervals (12AM–4AM, 4AM–8AM, 8AM–12PM, 12PM–4PM, 4PM–8PM, 8PM–12AM). We were interested in examining broader seasonal patterns of depredation (summer, monsoon and winter) and not just for individual months, hence each year was divided into 3 seasons of 4 months each (winter—November–February, summer i.e. February–June, monsoon i.e. June–November). We examined seasonal and temporal variation of attacks and difference in habitat types within the vicinity of predation sites using the chi-square test in R version 3.4.0. Statistical significance was *p* ≤ 0.05 for all analyses. All spatial analyses were performed with Arc GIS 10.3.3 and R 3.4.0.

The study areas were stratified into a scale of 5*5 i.e. 25 km^2^ which resulted to a total of 601 cells for North Bengal and 254 for Pauri Garhwal respectively. The cell size was selected based on ecological considerations to reduce chances of autocorrelation and identify landscape drivers of human–leopard conflicts. We generated a count statistic for the exact number of predation events for each cell. Cells where there were no attacks were assigned 0. To model the spatial spread and extent of livestock depredation we prepared both binary (presence-1 cells with at least one or multiple attacks, absence-0-no attacks) and count data (presence—exact number of attacks recorded and absence 0-no attacks). The number of cells on which leopard predation on livestock was recorded was 127 and 72 for North Bengal and Pauri Garhwal, respectively.

### Data preparation for spatial risk analysis

We identified a total of 5 major landscape features (Habitat, Water, Human presence and infrastructure, Distance to Protected Reserves and Altitude) for North Bengal and Pauri Garhwal (Table 3) based on their ecological importance to model predation risk.

1. Habitat: We hypothesized that predation risk by leopard will be higher in areas with forests, presence of riverine patches, water bodies. Area of land use types: We calculated landscape variables i.e. area under different land-use types and water sources such as area of riverine patches, water bodies from Forest type map of India (2014)^70^.
2. Water: Large predators are reported to kill prey in areas with availability of water^15, 20^, thus we hypothesized that availability of water will be a major driver of leopard predation on livestock. We calculated the length of rivers and area of river bodies from the Roads and Drainage layers obtained from Digital Chart of the World and Forest type map of India^70^.
3. Human presence and infrastructure: We hypothesized that leopards would avoid killing livestock in areas with increased human presence^71^. We extracted night light values using the 1,000-m spatial resolution night-time visible light data of India^72^. We calculated length of roads using the Roads and Drainage layers obtained from Digital Chart of the World.
4. Distance to Protected Reserves: We hypothesized that the probability of leopard killing livestock will be higher in areas closer to protected areas^17^. We calculated distance from protected areas (Protected Area Network of India) using the Euclidean distance tool for each cell.
5. Altitude: Considering that carnivores prefer to kill livestock in areas with gradient in altitude^47^, we hypothesized that predation risk by leopards will be higher in elevated regions. Hence, we generated the mean altitude value for each cell based on 90-m spatial resolution digital elevation maps 74.

**Table 3:** Major predictor variables considered for spatial analysis in the two study sites (25 km^2^).

Type of variable	Predictor variable	Unit	Range (North Bengal)	Range (Pauri Garhwal)	Resolution	Source
Habitat (landscape variables)	Area of non-forests	m^2^	0–2.3 × 10^7^	0–2.1 × 10^7^	30 m	FSI 2014
	Area of scrubland	m^2^	0–4.9 × 10^6^	0–5.4 × 10^6^	30 m	FSI 2014
	Area of moderate dense forests	m^2^	0–1.3 × 10^7^	0–2.2 × 10^7^	30 m	FSI 2014
	Area of very dense forests	m^2^	0–2.3 × 10^7^	0–1.5 × 10^7^	30 m	FSI 2014
	Area of open forest	m^2^	0–9.7 × 10^5^	0–2.2 × 10^7^	30 m	FSI 2014
Water	Area of river/water bodies	m^2^	0–5.6 × 10^5^	0–2.2 × 10^7^	30 m	Landsat 8TM
	Length of rivers	km	0–1.9	0.9.07	25 km^2^	Digital chart of the world
Human presence and infrastructure	Night light	Radiance	0–60	0–49	25 km^2^	Census India 2011
	Length of roads	km	0–4.75	0–8.92	25 km^2^	Digital chart of the world
Distance to protected reserves	Distance from protected areas	M	0–60,758	0–67,324	25 km^2^	Moef and CC, Govt. of India
Altitude	DEM	M	0–1,018	0–2,750	90 m	DEM

Once the cell files were finalized and compiled, we clipped all ecological variables to 25 km^2^ cells. We omitted redundant correlated variables ≥ 0.70^74^ based on Pearson correlation coefficient values calculated using R version 3.4.0. Area of moderate dense forests was positively correlated with the area of very dense forests in North Bengal (value 0.76) and hence it was excluded from the analysis. Length of river was also positively correlated with length of roads (value 0.8) and hence it was also removed from the analysis. Altitude was positively correlated with distance to protected areas in Pauri Garhwal (value 0.76) and hence it was excluded from the analysis.

We used 4 analytical approaches to model probability of livestock depredation by leopard. In the 1st step we evaluated spatial autocorrelation among livestock kills within the cells using function moran.test (Moran’s I) in package (spdep)^75^ in R 3.4.0. In the 2nd and 3rd approach we used generalized linear models (GLMs) with binomial and poisson structures to quantify the effect of landscape features (area of habitat types, availability of water, human presence and infrastructure, distance to protected areas and altitude) on livestock predation. All the predictor variables considered for the analysis were continuous in nature. We used a priori candidate models and ranked them based on AIC, AICc values^76^. Models with the lowest AICc values were considered the best or dominant model and the output (coefficients and estimates) explained the probability of livestock predation by leopards within IHR. Based on the results of the dominant model or the model averaged coefficients, we generated conflict hotspots for both the study sites. We used coefficients of the best model (binomial structure) or averaged all candidate models (GLM with binomial structure) to estimate probability of livestock depredation for each cell (25 km^2^) using the equation p (x) = exp (z)/ (1 + exp (z)) and generated human–leopard conflict hotspots in Arc GIS 10.3^77^. We generated ROC curve and AUC values to predict reliability of the dominant models using package ROCR^78^ in R 3.4.0. Since predictor variables between the two study sites were not normally distributed, we compared the identical landscape features using nonparametric Wilcoxon Signed-Rank Test in R 3.4.0.

In the 4th step, we used the predictor variables of the dominant models to calculate conditional inference (CTREEs), as prescribed in the R-package “partykit”^79^. This method was adopted to obtain threshold values for the significant variables for conflict mitigation recommendations. Trees based on maximally selected rank statistics were fitted using the Bonferroni correction for multiple testing and a minimum sum of weights. In addition, univariate trees were fitted for variables with a significant split in the multivariate tree. The results of our two analytical approaches (regression and ctree) are similar and provide an overall understanding of landscape features prone to livestock predation in accordance with the behavior of common leopards. Both analytical methods are based on a maximum likelihood approach and when interpreted together provide meaningful results. The GLM models computes probabilities of an event based on a logistic regression framework while the CTREE uses a machine learning classification approach and assigns values to predefined categories. The decision tree approach is a non-parametric approach which helps simplify complex relationships between dependent and predictor variables.

## Supplementary information

Supplementary Information
