# Supplementary material for: Landscape predictors of human–leopard conflicts within multi-use areas of the Himalayan region
Source: Sci Rep. 2020 Jul 7;10:11129. doi: 10.1038/s41598-020-67980-w (PMC7341814; doi:10.1038/s41598-020-67980-w)
Supplement: Supplementary file 1 — Supplementary Information [file 41598_2020_67980_MOESM1_ESM.docx]

**Landscape predictors of human-leopard conflicts within multi-use areas of the Himalayan Region**

**Dipanjan Naha^1^, Suraj Kumar Dash^1^, Abhishek Chettri^1^, Pooja Chaudhary^1^, Gaurav Sonker^1^, Marco Heurich^2^, Gopal Singh Rawat^1^, Sambandam Sathyakumar^1*^**

**^Supplementary Information Appendix 1^**

**Datasheet for livestock depredation by common leopard in IHR (2015-2018)**

Name of District:

Name of village/locality:

Livestock species killed:

Number of livestock killed:

Probable age of livestock killed:

GPS location of kill site:

Elevation of kill site:

Time of livestock kill:

Season of livestock kill:

Number of houses present within 50 meters’ radius of kill site:

Vegetation type of kill site (50-meter radius):

Whether livestock was supervised:

Any additional comments:

**Supplementary Table S1 Summary of the dominant generalized linear model with binomial structure for probability of livestock predation by leopard within North Bengal landscape**

| **Coefficients** | **Estimate** | **Standard error** | **Z value** | **Probability** |
| --- | --- | --- | --- | --- |
| Intercept | -1.950 | 0.580 | -3.359 | 0.0007 |
| Altitude | -5.263e-03 | 0.001 | -5.234 | 1.66e-07 |
| Nightlight | -7.447e-02 | 0.031 | -2.421 | 0.0154 |
| Distance from PA | -1.703e-04 | 2.729e-05 | -6.243 | 4.29e-10 |
| Area of Water/Riverine patches | -1.607e-06 | 5.624e-07 | -2.858 | 0.00426 |
| Area of Scrubland | 1.879e-06 | 5.664e-07 | 3.318 | 0.00091 |
| Area of Non Forest | 7.814e-08 | 3.224e-08 | -2.424 | 0.01535 |
| Area of Open Forest | 2.558e-07 | 3.786e-08 | 6.756 | 1.42e-11 |
| Area of Very Dense Forest | 1.978e-07 | 4.321e-08 | 4.577 | 4.71e-06 |

**Supplementary Table S2 Summary of the dominant generalized linear model with poisson structure for probability of livestock predation by leopard within North Bengal landscape**

| **Coefficients** | **Estimate** | **Standard error** | **Z value** | **Probability** |
| --- | --- | --- | --- | --- |
| Intercept | -1.804 | 4.353e-01 | -4.145 | 3.40e-05 |
| Altitude | -2.596e-03 | 7.127e-04 | -3.642 | 0.000270 |
| Nightlight | -3.945e-02 | 2.119e-02 | -1.861 | 0.062683 |
| Distance from PA | -1.070e-04 | 1.776e-05 | -6.026 | 1.68e-09 |
| Area of Water/Riverine patches | -9.112e-07 | 4.327e-07 | -2.106 | 0.035219 |
| Area of Scrubland | 2.498e-07 | 1.309e-07 | 1.909 | 0.056304 |
| Area of Non Forest | 4.526e-08 | 2.438e-08 | 1.856 | 0.063409 |
| Area of Open Forest | 1.340e-07 | 2.369e-08 | 5.657 | 1.54e-08 |
| Area of Very Dense Forest | 9.387e-08 | 2.501e-08 | 3.753 | 0.000175 |

**Supplementary Table S3 Summary of the dominant generalized linear model with binomial structure for probability of livestock predation by leopard within Pauri Garhwal landscape**

| **Coefficients** | **Estimate** | **Standard error** | **Z value** | **Probability** |
| --- | --- | --- | --- | --- |
| Intercept | -9.149 | 2.015 | -4.542 | 5.58e-06 |
| Distance from PA | 3.110e-05 | 1.187e-05 | 2.619 | 0.008808 |
| Area of Scrubland | 5.579e-07 | 2.423e-07 | 2.302 | 0.021331 |
| Area of Non Forest | 3.384e-07 | 7.463e-08 | 4.535 | 5.77e-06 |
| Area of Open Forest | 4.620e-07 | 1.198e-07. | 3.857 | 0.000115 |
| Area of Moderate Dense Forest | 2.954e-07 | 8.896e-08 | 3.320 | 0.0009 |

**Supplementary Table S4 Summary of the dominant generalized linear model with poisson structure for probability of livestock predation by leopard within Pauri Garhwal landscape**

| **Coefficients** | **Estimate** | **Standard error** | **Z value** | **Probability** |
| --- | --- | --- | --- | --- |
| Intercept | -8.431 | 1.950 | -4.324 | 1.53e-05 |
| Nightlight | 2.558e-02 | 1.002e-02 | 2.554 | 0.0107 |
| Distance from PA | 2.587e-05 | 4.393e-06 | 5.889 | 3.88e-09 |
| Area of Scrubland | 4.085e-07 | 9.706e-08 | 4.209 | 2.57e-05 |
| Area of Non Forest | 3.323e-07 | 7.828e-08 | 4.245 | 2.18e-05 |
| Area of Open Forest | 4.953e-07 | 8.196e-07. | 6.044 | 1.50e-09 |
| Area of Moderate Dense Forest | 3.334e-07 | 7.874e-08 | 4.234 | 2.30e-05 |

**Supplementary Table S5 Wilcoxon Signed Rank Sum Test statistics showing the test coefficient (W) and P-value (P) for landscape feature predictor variables between kill sites of the two study regions at a spatial scale of 25 km².**

| **Predictor Variable** | **W** | **P** |
| --- | --- | --- |
| Area of Very Dense Forest | 55179 | 8.147e-12 |
| Area of Moderate Dense Forest | 16963 | < 2.2e-16 |
| Area of Open Forest | 92719 | 6.789e-07 |
| Area of scrubland | 65678 | 2.624e-07 |
| Area of Non Forest | 94949 | 1.672e-08 |
| Area of water/Riverine patches | 95532 | 6.827e-10 |
| Length of Road | 35600 | < 2.2e-16 |
| Length of River | 24430 | < 2.2e-16 |
| Distance from PA | 71253 | 0.124 |
| Nightlight | 92562 | 9.875e-08 |
| Altitude | 5901.5 | < 2.2e-16 |
